# Inaugural ‘One Health Day’ organised on 3 November 2016

**DOI:** 10.2807/1560-7917.ES.2016.21.44.30386

**Published:** 2016-11-03

**Authors:** Eurosurveillance editorial team

**Affiliations:** 1 European Centre for Disease Prevention and Control (ECDC), Stockholm, Sweden

The very first ‘One Health Day’ will be organised on 3 November 2016 across the globe. It is held to promote a change in the assessment of global health challenges and how to address them, and aims to ‘bring global attention to the need for One Health interactions’. It is intended as ‘a day of declaration and action wherever possible to bring global attention to the crucial need and benefits of using trans-disciplinary approaches to complex challenges involving animals, people, and planetary ecosystems’.

One Health Day is organised by three international One Health groups: the One Health Commission, the One Health Initiative Autonomous pro bono Team and the One Health Platform Foundation.

The ‘One Health Initiative’ was first started in April 2007 when the American Veterinary Medical Association formed the One Health Initiative Task Force. The link between human infectious diseases and zoonoses is frequently underlined on the many sites forming the One Health initiative as ‘nearly 75 percent of all emerging human infectious diseases in the past three decades originated in animals’.

Read more about One Health here.

